# Physical Health Monitoring in Waverley Community Mental Health Recovery Service (CMHRS)

**DOI:** 10.1192/bjo.2024.354

**Published:** 2024-08-01

**Authors:** Steve Calvosa

**Affiliations:** Surrey and Borders Partnership Foundation Trust, Guildford, United Kingdom

## Abstract

**Aims:**

To audit the recording of physical health parameters for the clients of Waverley Community Mental Health Recovery Service (CMHRS).

To ensure Trust and NICE guidelines are met for monitoring of:

1. Psychiatric drug prescribing.
2. Psychiatric disease monitoring.
3. Past medical history and biophysical parameters relevant to prescribing decisions.

To develop a clinical review process for the clients to ensure that physical health parameters are monitored longitudinally.

**Methods:**

A random sample of 100 patients from Waverley CMHRS was analysed. The data was collected between November 2022 and January 2023. The process involved establishing the cohort, dividing the caseload for review, and applying an audit questionnaire. The questionnaire was applied to both SystmOne Electronic Patient Records and GP Shared Care Records to assess compliance with physical health monitoring in both secondary and primary care. All data collected were compiled onto an Excel Spreadsheet. The level of compliance for monitoring of each parameter was calculated and audited against Trust and NICE guidance.

**Results:**

For secondary care:

1. Compliance with physical health monitoring requirements is consistently low.
2. Higher levels of compliance (>50%) for height, weight, Audit C (Alcohol), Smoking status.
3. Lowest compliance levels observed for: blood tests, ECG request, substance misuse status, sleep, medication side effects.
4. Evidence of a comprehensive physical health review was found in 1% of patients.

For primary care:

1. 95% of patients from our sample consented to giving access to their Shared Care Record.
2. Compliance with physical health monitoring requirements in primary care was higher.
3. Compliance was particularly high (> 87%) for: height, weight and BMI, BP, evidence of alcohol monitoring, evidence of smoking monitoring.
4. Smoking monitoring is the parameter with the highest level of compliance (95%).
5. Parameters are monitored more regularly.

**Conclusion:**

The audit identified gaps in the documentation and assessment of physical health parameters within Waverley CMHRS. Compliance with monitoring requirements was significantly lower in secondary care, highlighting the need for intervention. Conversely, primary care demonstrated higher adherence to monitoring guidelines. The results show deficiencies in physical health monitoring that need to be addressed to ensure comprehensive psychiatric care.

The project was crucial in optimizing physical health monitoring within Waverley CMHRS. Recommendations include targeted training, improved communication between primary and secondary care, and the designation of physical health coordinators. An action plan was developed with assigned responsibilities and a timeline for implementation. A re-audit will follow to assess the impact of implemented changes.

